# Association of high organochlorine pesticide serum levels with oxidative stress in intestinal metaplasia and gastric cancer

**DOI:** 10.1016/j.heliyon.2024.e41599

**Published:** 2025-01-03

**Authors:** Gholamreza Asadikaram, Mohammad Reza Ashrafi, Sodaif Darvish Moghaddam, Moslem Abolhassani, Fatemeh Bagheri

**Affiliations:** a Applied Cellular and Molecular Research Center, Kerman University of Medical Sciences, Kerman, Iran; b Neuroscience Research Center, Institute of Neuropharmacology, Kerman University of Medical Sciences, Kerman, Iran; c Department of Clinical Biochemistry, Afzalipur School of Medicine, Kerman University of Medical Sciences, Kerman, Iran; d Gastroenterology and Hepatology Research Center, Kerman University of Medical Sciences, Kerman, Iran; e Pathology and Stem Cell Research Center, Afzalipour School of Medicine, Kerman University of Medical Sciences, Kerman, Iran

**Keywords:** Organochlorine pesticides, Oxidative stress, Gastric cancer, Intestinal metaplasia, Functional dyspepsia

## Abstract

It has been revealed that certain organochlorine pesticides may increase the risk of gastric cancer incidence among farm workers. This research aimed to assay levels of 7 different organochlorine pesticides in the serum of functional dyspepsia, intestinal metaplasia and gastric cancer patients, along with measuring oxidative stress parameters compared to the control group and their associations. The levels of organochlorine pesticides in the serum of gastric cancer (n = 34), intestinal metaplasia (n = 8), functional dyspepsia patients (n = 48) and control group (n = 46), were measured by gas chromatography equipment. Oxidative stress parameters and erythrocyte acetylcholine esterase activity were also measured in these individuals. The levels of some organochlorine pesticides in the serum of gastric cancer patients were significantly higher than those in the control group. Moreover, the profiles of organochlorine pesticide concentrations were different in gastric cancer, intestinal metaplasia and functional dyspepsia patients. Overall, oxidant parameters were significantly higher in gastric cancer and intestinal metaplasia patients than those in functional dyspepsia patients and the control group, except in the case of malondialdehyde. The antioxidant and acetylcholine esterase enzyme activities were significantly higher in the control group than in other groups. Our results showed that progression from functional dyspepsia towards intestinal metaplasia and gastric cancer was associated with elevations in organochlorine pesticide serum levels, which had an association with the induction of oxidative stress and the reduction of antioxidant and acetylcholine esterase enzyme activities.

## 1 Introduction

In the modern world, cancer is the leading cause of death in 91 countries [1]. Gastric cancer (GC) was the fifth most commonly diagnosed carcinoma and the third cause of cancer death worldwide in 2018 [1]. Functional dyspepsia (FD) is a non-ulcer upper gastrointestinal symptom that has a prevalence of 11–29.2 % globally [2]. Gastritis is a asymptomatic disorder, which has been implicated in the development of gastric mucosa atrophy [3]. Symptoms of Gastritis may even progress to intestinal metaplasia (IM) and then GC [3].IM has been introduced as a pre-cancerous lesion, which may increase the GC risk by 6fold [4]. Elevated Oxidative stress (OS) (an imbalance between OS and antioxidant responses in favor of OS) has been indicated as an index in the pathology of different diseases, including FD [5] and cancer [6]. In previous studies, a relation between the total antioxidant capacity of plasma and the risk of GC has been documented [[7], [8], [9]]. Acetylcholine esterase (AchE) is the enzyme responsible for the hydrolysis of acetylcholine in neurons and other tissues, including the upper gastrointestinal regions [10]. AchE activity has been revealed to be reduced in cancerous gastric tissues, and even elevated ectopic activity has therapeutic effects [10]. In another study, Zhi Gu and colleagues surveyed the serum activity of AchE in GC patients in comparison with the normal control group and concluded that serum AchE activity has a close association with GC incidence [11]. The inhibition of AchE activity could have vast effects on improving the symptoms of FD patients [12]. Pesticides are a group of toxic chemicals that are released into the environment by humans for the elimination of pests, especially in agriculture [13]. Based on their chemical structures, pesticides are divided into three classes: organochlorine, organophosphorus and inorganic [13]. These compounds could lead to harm to human health via occupational exposure, food and liquid contamination, and even air pollution [13]. Exposure to pesticides may result from working at the place of production, transportation and preparation of these chemicals or application of these compounds in agricultural occupations [14]. Furthermore, pesticides could be resolved by soil and agricultural products, are settled into the groundwater and accordingly to the rivers and seas, thus entering the food chain and human body. Thus, the origin of OCPs in environment and body is difficult to detect [15]. Pesticides could cause a diverse range of diseases such as cancer, diabetes, asthma and Parkinson's disease [13]. Organochlorine pesticides have been documented to have a strong association with the incidence of GC [16], breast cancer [17], colon cancer [18] and bladder cancer [19]. In this study, serum levels of 7 derivatives of OCPs (α-HCH, β-HCH, γ-HCH, 2,4-DDE and 4,4-DDE, 2,4-DDT, 4,4-DDT) in the FD, IM, GC and control groups were investigated. In addition, the enzyme activities of AchE in erythrocytes, paraoxonase-1 (PON1), glutathione peroxidase-3 (GPx3), superoxide dismutase-3 (SOD3), catalase (CAT), and oxidative stress (OS) markers, including malondialdehyde (MDA), nitric oxide (NO), and protein carbonyl (PC) were evaluated.

The purpose of the study was to determine whether elevated serum OCP levels are associated with the induction of OS and alterations in antioxidant enzyme activities contributing to the progression from FD toward IM and GC for the first time in the southeast of Iran. The rationale behind of this study comes from our previous research that have been revealed that OCPs present at least in some parts of the studied area [[17], [18], [19]].

## 2 Material and methods

### 2.1 Subjects

Our case-control study was performed on the patients diagnosed with FD, IM with chronic gastritis, and GC (intestinal type), along with our normal control (Ctrl) group (healthy individuals). Samples were collected from Kerman Afzalipour Hospital from July 2018 to April 2020. The study groups included newly diagnosed FD patients (n = 48), IM with chronic gastritis patients (n = 8), GC patients (n = 34) and healthy Ctrl individuals (n = 46). The diagnosis of these patients was based on the clinical findings of gastrointestinal subspecialists and the pathology reports. All participants were new cases and had no history of previous diseases in this study. They were not prescribed any drug, supplement or narcotic. All subjects signed and read consent forms willingly to consent to this research project. All the patients were diagnosed with primary GC, and patients with a prior history of GC and other cancers metastasized from elsewhere to the stomach were excluded from our study. The declaration of Helsinki standards was applied to physicians and patients in our study. This study was approved by the ethics committee of Kerman University of Medical Sciences, Kerman, by code number IR. KMU. REC.1398.335.

### 2.2 Samples collection

10 mL of venous blood samples were collected from each individual. A total of 0.5 mL of the blood samples was transferred into the EDTA-treated tubes (for the erythrocyte cholinesterase activity assay), and the remnants were placed in the tubes in the absence of anticoagulation substances. The blood samples were then centrifuged to separate the serum and plasma (at 3000 rpm for 10 min). Plasma and serum samples were transferred to sterile specimen containers and stored at −70 °C for future analysis [19].

### 2.3 Biochemical parameters

Serum levels of total cholesterol (TC), total protein, triglycerides (TG) and high-density lipoprotein-cholesterol (HDL-c) were determined by the standard kits [Pars Azmoon, Tehran, Iran] in standard laboratory settings by an autoanalyzer (Selectra-XL, vital science; Netherlands). The Friedewald equation was used for the calculation of Low-density lipoprotein-cholesterol (LDL-c) [19].

### 2.4 AchE activity assay

Acetylcholine iodide, 5, 5-dithio-bis-2-nitrobenzoic acid (DTNB) and hyamine 1622 were purchased from Sigma (Saint Louis, MO, USA). The Erythrocyte AChE activity was calculated in all the patient samples using Ellman's modified procedure [20]. First, 6 mL of distilled water was used to dilute the 100 μl of erythrocytes, which were washed prior with the normal saline solution. The reaction buffer (a mixture containing 0.28 mmol of DNTB, 3.2 mmol of Acetylcholine iodide, and 20 μm quinidine sulfate) was used for incubating the 100 μl of the diluted sample at 37 °C for 10 min. Finally, 1 mL of Hyamine 1622 was added to the solution to stop the reaction. 5-thio-2-nitrobenzoic acid (with a maximum absorbance of 440 nm) was the end product of the reaction of thiocholine and DTNB chromophore [18,19,21].

### 2.5 MDA measurement

The thiobarbituric acid (TBA) assay method was used for measuring the MDA as an index for lipid peroxidation. In the presence of the trichloroacetic acid (TCA (15%w/v))-TBA (0.375 % w/v)-Hydrochloric acid (HCL (0.25 N)) reagent, as a result of MDA reaction with the TBA, a pink color was formed and the absorbance was measured at 535 nm [22].

### 2.6 Total antioxidant capacity serum level assay

The ferric-reducing ability of the plasma (FRAP) was measured by Benzie and Strain's proposed method [23]. 5 μL of plasma was mixed with 70 μL of FRAP reagent. The mixture was then incubated at 37 °C for 5 min, and the absorbance was acquired at 593 nm. The FRAP values expressed as micromoles per liter (mM) and a standard curve that exhibited millimoles of Fe^2+^ in the absorbance were applied to obtain these values [18,19,21].

### 2.7 The determination of SOD3 activity

The SOD3 activity was determined using RANSOD (RANDOX) kit (UK) protocol. SOD3 activity was measured by the calculation of reduction extent which occurred in the presence of NBT-diformazan in plasma samples at a wavelength of 560 nm [23].

### 2.8 The determination of GPx3 activity

GPx3 assay was determined based on the method described by Paglia and Valentine by RANSOD (RANDOX) kit (UK) [23]. The Randox Glutathione Peroxidase Assay Kit measures GPx3 activity indirectly by a coupled reaction with glutathione reductase (GR) which consequently causes oxidation of NADPH to NADP+ and Absorbance was obtained at 340 nm [23].

### 2.9 Protein carbonyls (PC) assay

The measurement of PC following its covalent reaction with 2,4-dinitrophenylhydrazine (DNPH) was proposed by Levine et al. [23]. This procedure was performed by incubation of 40.5 mg protein that are modified by oxidation with 10 mM DNPH for 1 h. Proteins were precipitated by adding 20 % TCA. The precipitates were then washed three times with ethanol–ethyl acetate (1:1) and finally dissolved in 6M guanidine. The last step was the spectrophotometry measurement of the 2,4-dinitrophenyl (DNP) hydrazones at 370 nm [18].

### 2.10 Measurement of the arylesterase activity of PON1

Phenylacetate (a substrate for arylesterase activity) was purchased from Sigma Chemical Co. (Saint Louis, MO, USA). Serum arylesterase activity of PON1 was measured by Rate of phenylacetate hydrolysis according to the procedure suggested by Bobin-Dubigeon et al. [23]. The reaction mixture was 2 mM of Phenylacetate, 2 mM CaCl_2_ (Merck, Darmstadt, Germany) and 10 μl of serum in 100 mM Tris-HCl (Merck, Darmstadt, Germany) (pH = 8.0). The mixture was then incubated at 37 °C for 3 min. Finally, the phenylacetate hydrolysis rate was measured at 270 nm [19].

### 2.11 Determination of NO concentration

The nitric oxide (NO) level in the serum was evaluated using the Griess method. The serum deproteinization was initially performed by ZnSO 4 in the presence of 0.3 M NaOH. Vanadium (III) chloride (VaCl3) (the active catalyst for nitrate to nitrite conversion) and the Griess Reagent (2 % sulphanilamide in 5 % Phosphoric acid and 0.1 % N- (1-Naphthyl ethylendiamine dihydrochloride (NEDD) in deionized water) were then mixed with the protein-free serum. The mixture was incubated at 37 °C for 30 min and then the optical density (OD) of the mixture was measured at 540 nm [24].

### 2.12 Measurement of OCPs

The standard for organochlorine pesticides (OCP) components, including α-HCH, β-HCH, γ-HCH, 2,4-DDT, 4,4-DDT, 2,4-DDE and 4,4-DDE (4,4-dichlorobenzophenone (DBP)) was obtained from Ehrenstorfer Company (Germany). N-hexane, anhydrous sodium sulfate and Ethyl acetate were purchased from Merck (Germany) and sulphuric acid was obtained from Scharlab (Spain). A gas chromatographic analyzer (Agilent 7890A, USA) coupled with a flame ionization detector (GC-FID) was used for detection and measurement of components of the serum OCPs for all samples. Zumbado et al. (2005) suggested a modified method that was used for identifying OCP residues [25] as described previously [[17], [18], [19]]. First, the internal standard (4, 4-Dichlorobenzophenone (DBP)) was mixed with 0.5 mL of serum. Sample extraction was repeated twice with 2 mL of hexane. 200 μL of concentrated sulphuric acid was then added for separation of the organic fraction of the serum. Then 100 mg of anhydrous sodium sulfate was used to dehydrate this organic fraction. The concentrated organic layer was centrifuged and then air-dried at room temperature for 48 h. Finally, 100 μL of Ethyl acetate was added to each sample to resolve the extracted OCPs. Gas Chromatography with a flame ionization detector and capillary columns (HP-5) was used as an analytical method for the identification of OCPs. The retention time (qualitative analysis), peak area (quantitative analysis), and internal standard were used to calculate the serum levels of OCPs. Therefore, a set of OCP standard solutions with determined concentrations (0.78, 1.56, 3.12, 6.25, 12.5, 25, 50, 100, 200 and 400 μg/mL) were prepared and then equal values of the DBP internal standard (100 μg/mL) added to each OCP standard solution. The peak areas of the OCP standards and DBP were then calculated and seven calibration curves were drawn for seven OCP compounds, representing the ratio of the OCP standard's peak area to the internal standard versus the concentration ratio were created for seven OCP compounds. The ratio of the peak area was then used to determine the areas of the OCPs and internal standards for unidentified samples. OCP concentrations were ascertained in the last stage using OCP standard curves. To guarantee precise OCP quantification, quality assurance and control, or QA/QC, were upheld. In addition to field blanks and equipment blanks, each sample was examined in triplicate. All the analytical results reported are the average of three values, so the method's performance can be evaluated. In this regard, a set of pesticides' standard solutions with known concentrations (0.05, 0.1, 0.5, 0.75, 1, 2, 4, 8, 16, 25, 50 and100 μg/L) were spiked in the pooled sample, and the calibration curves were obtained. Procedure blanks were prepared using ethyl acetate and routinely analyzed to check for inlet, column, and detector contamination during extraction and injection, to examine the cross-contamination and to monitor the background contamination of the instrument [[17], [18], [19]]. The analytical limit of detection was estimated to be 0.7 μg/mL for α-HCH, β-HCH, γ-HCH, 2,4-DDE and 4,4-DDE and 3 μg/mL for 2,4-DDT, 4,4-DDT [[17], [18], [19]].

### 2.13 Statistical analysis

Mean ± standard error of the mean (mean ± SEM) was used to represent all continuous variable data, numbers (percentages) and categorical variables. Data distribution was determined by the Kolmogorov-Smirnov test. To exhibit the differences between groups, one-way Analysis of Variance (ANOVA)/Kruskal-Wallis with post-hoc Tukey/Mann-Whitney U tests and Chi-square/Fisher's exact tests were used. The correlations between continuous variables were manifested using Pearson and Spearman rho correlation coefficients. SPSS software version 22.0 for Windows (SPSS Inc., Chicago, IL) was used for the statistical analyses. P values < 0.05 were considered statistically significant.

## 3 Results

### 3.1 AchE and OS parameters

**Fig. 1:**
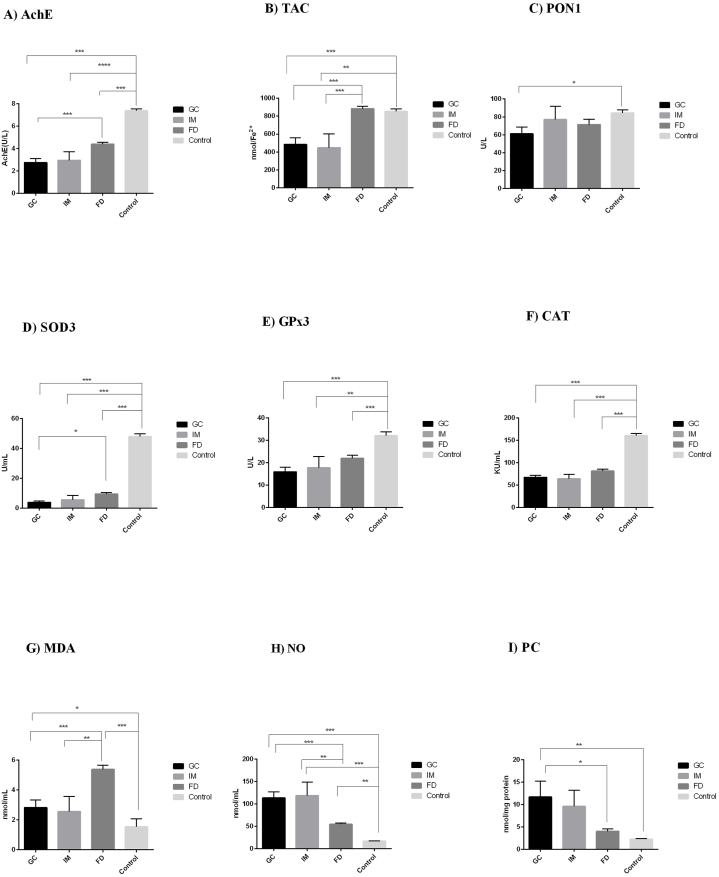
Parameters of oxidative stress and AchE activity between GC, IM, FD and Ctrl groups have been compared here. As indicated, A. AchE activity is significantly lower in the GC and IM groups than in the FD and Ctrl groups. B. TAC is significantly lower in GC and IM compared to other groups. C.PON1 activity is lower in GC compared to the Ctrl group. D.GC and IM groups have significantly lower activity of SOD3 in comparison to other groups. E. Ctrl group has the highest activity of GPx3 compared to other groups. F. CAT activity in the Ctrl group is significantly higher than others. G. MDA oxidant has the lowest amount in the Ctrl and the highest in the FD group. H. Levels of NO as reactive nitrogen species are significantly highest among GC and IM and lowest in the Ctrl group. I. levels of PC as an index of oxidation are significantly higher in GC than in FD and Ctrl groups. ∗ Indicates *p*-values less than 0.05, ∗∗ shows *p*-values less than 0.005 and ∗∗∗ is indicative of *p*-values less than 0.0005. GC: gastric cancer patients; IM: intestinal metaplasia along with chronic gastritis patients; FD: functional dyspepsia patients; Ctrl: control group.

As shown in Fig. 1, AchE activity was significantly lower in the GC, FD and IM groups in comparison to the Ctrl group and the FD group had higher AchE activity than the GC group. The FD group had insignificantly higher levels of AchE activity than the IM group. In our study, TAC in serum was lower in GC patients and IM in comparison to the FD and Ctrl groups but there was no significant difference between the GC and IM groups or between the FD and Ctrl groups. In the case of GPx3, the activity of this enzyme was significantly lower in the GC, IM and FD groups as compared to the Ctrl group and the FD group had insignificantly higher activities compared to the IM and GC groups, and the IM had insignificantly higher activities compared to the GC group. PON1 enzyme activity was significantly higher in the Ctrl group in comparison with the GC group. The activity of PON1 was insignificantly higher in the IM group compared to the FD and GC groups, and the activity of this enzyme was insignificantly higher in the FD group compared to the GC group. SOD3 enzyme activities were significantly higher in the Ctrl group than in the GC, IM and FD groups and the FD group had significantly higher activities of this enzyme compared to the GC group. The IM group had insignificant higher activities of SOD3 in comparison with the GC group. In our study, the CAT activity of the Ctrl group was significantly higher than that of the GC, IM and FD groups. In the FD group CAT activity was insignificantly higher than those in the IM and GC groups. Our results showed that MDA levels were significantly higher in the FD group as compared to GC, IM and Ctrl and the GC group had significantly higher levels of MDA in comparison with the Ctrl group. The MDA levels in the IM group were insignificantly higher than those in the Ctrl and lower than those in the GC group. Our results revealed that NO levels in the GC and IM groups were significantly higher than the FD and the Ctrl groups and in the FD group, they were higher than the Ctrl group. The IM group had insignificantly higher levels of NO than the GC group. PC was significantly higher in the GC group as compared with the FD and Ctrl groups. The IM group had insignificantly higher levels of PC compared to the Ctrl and FD groups and lower levels in comparison with the GC group.

### 3.2 Serum OCP levels

**Fig. 2:**
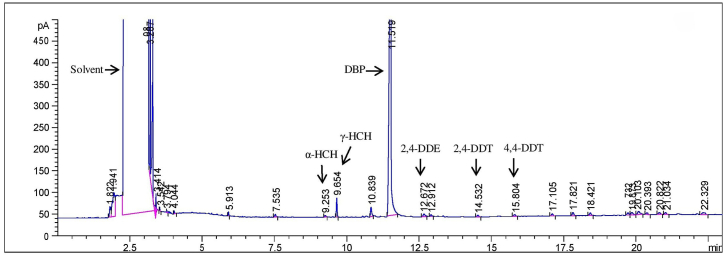
A gas chromatogram showing peaks of serum OCPs and internal standards in a GC patient. α-HCH peak at 9.2, β-HCH peak at 9.5, γ-HCH peak at 9.6, 2,4-DDE peak at between 12.3 and 12.6, 4,4-DDE peak at between 13.3 and 13.6, 2,4-DDT at between 14.5 and 14.7 and 4,4-DDT peak at between 15.5-15.8 min were observed. The peak at 11.5 min is the internal standard DBP(4,4′dichlorobenzophenon).

A chromatogram indicating the OCP levels in a GC patient has been shown in Fig. 2. As indexed in Table 2, in our study, levels of γ-HCH (*p* = 0.042), 2,4-DDE (*p* = 0.022), 2,4-DDT (*p* = 0.025) and 4,4-DDT (*p* = 0.045) were significantly higher in the GC group in comparison to the Ctrl group. Other OCPs were higher in the GC group compared with the Ctrl group, although not significantly. Additionally, levels of α-HCH (*p* = 0.014), γ-HCH (*p* = 0.041) and 2,4-DDE (*p* = 0.026) were significantly higher in the GC group as compared to the FD group. The GC group had higher levels of the remaining 4 OCPs compared to the FD group insignificantly. The IM group had significantly higher levels of α-HCH (*p* = 0.017) and β-HCH (*p* = 0.001) compared to the FD group. 4,4-DDE, 2,4-DDT and 4,4-DDT levels were insignificantly higher in the FD group in comparison with that of the IM group. Levels of β-HCH in the IM group were significantly higher than the Ctrl and GC groups (both had *p* = 0.001). α-HCH and 2,4-DDE levels were insignificantly higher in the IM group in comparison with the GC group. Levels of γ-HCH, 4,4-DDE, 2,4-DDT and 4,4-DDT were insignificantly higher in the GC group compared to that of the IM group. There was no significant difference in pesticide levels between the FD and the Ctrl groups, although the FD group had insignificantly higher levels of α-HCH, β-HCH, 4,4-DDE, 2,4-DDT and 4,4-DDT compared to the Ctrl group and levels of the other 2 OCPs were insignificantly higher in the Ctrl group. Logistic regression analysis showed that 2,4-DDT (OR = 1.89; 95%CI = 1.193–2.995) and 4,4-DDT (OR = 1.916; 95%CI = 1.214–3.023) could increase the risk of GC.

### 3.3 Spearman rho correlation analysis

As shown in Table 3, using Spearman rho analysis, correlations between different parameters, including OCP exposure and OS, have been examined. Based on these results, AchE activity is shown to have reverse relations with γ-HCH, 2,4-DDE, 2,4-DDT and 4,4-DDT. TAC was indicated to be reversibly associated with γ-HCH and 2,4-DDE. SOD3 has been shown to have reverse relationships with γ-HCH, 2,4-DDE, 2,4-DDT and 4,4-DDT. GPx3 is designated to have reverse associations with α-HCH, γ-HCH, 2,4-DDE, 2,4-DDT and 4,4-DDT. CAT is indicated to have reverse associations with γ-HCH, 2,4-DDE, 2,4-DDT and 4,4-DDT. NO is specified to have direct relationships with α-HCH, β-HCH, γ-HCH, 2,4-DDE, 2,4-DDT and 4,4-DDT. MDA was indicated to be directly associated with 2,4-DDT and 4,4-DDT. PC was shown to be directly associated with α-HCH, γ-HCH and 2,4-DDE. The correlations between other parameters were not significant, as shown in Table 3.

## 4 Discussion

The purpose of this study was to measure serum OCP levels and survey its association with the induction of OS in GC, IM and FD patients in comparison with the Ctrl group. To the best of our knowledge, there has not been an experiment to examine these parameters in the above-mentioned patient groups. Previous research has measured only limited OCPs, such as 2,4-dichlorophenoxyacetic acid, chlordane, and propargite, rather than the seven OCPs examined in this study. Mills and colleagues have concluded that OCPs could increase the risk of GC among farmworkers [16]. The present study, reveals a strong association between elevated serum of some OCP levels and OS induction in GC and IM patients in the general population of southeastern Iran.

Significantly higher OCP levels in GC and IM patients suggest a link between exposure to these chemicals and disease progression. The sources of potential exposure may include occupational hazards, the consumption of contaminated food or water, and environmental factors. OCPs have been reported to exhibit lipophilic properties and have a long half-life which contribute to their accumulation in adipose tissue, leading to prolonged retention in the body, which increases the risk of developing malignancies [19].

Overall**,** the total antioxidant capacity of patients would be lower than that of normal individuals. However the absence of a significant difference between the above-mentioned groups could be attributed to the possible consumption of anti-oxidant agents or the role of other oxidant/anti-oxidant system components which were not measured in this study [7,9]. In the case of comparison between GC and IM groups, anti-oxidant capacities have been critically decreased in these patient groups almost equally, indicating that additional genetic mutations may drive the progression from IM to GC [26]. Higher activities of AchE in FD patients may contribute to some symptoms of this disease [12]. The reduction in AchE activity in GC patients aligns with the evidence of pesticide-induced toxicity and increased OS markers [27]. These findings are in agreement with prior studies that have reported lower AchE levels in the serum [11] and gastric tissue of GC patients [10]. According to the fact that AchE is known to stimulate antioxidant enzymes, such as SOD, its reduced activity could exacerbate OS and promote carcinogenesis [18]. The observed decrease in GPx3 activity in GC patients is in accordance with the increased production of ROS typical of cancer [28]. Similarly, the lower PON1 activity observed in GC patients is consistent with previous findings [29].

Previous studies showed lower SOD activity in cancer patients [30] and higher SOD activity in the other one [31] compared to the Ctrl group. These differences could be interpreted by heterogeneity of cancer cells which suggests even the same kind of cancer cell could exhibit different molecular signatures [32]. Ethnic background, dietary habits, and chemical exposure, including OCPs, may further contribute to these discrepancies [18]. Moreover, pesticides including hexachlorocyclohexanes (HCHs) have been revealed to reduce SOD enzyme activity, consistent with our results [33].

While prior studies have not directly compared CAT activity in the serum of GC patients and healthy individuals, evidence from GC tissue samples suggests higher CAT activity in cancerous tissues compared to adjacent normal tissue [34]. Our results imply that in cancer patients, antioxidant systems may have diminished ability to neutralize hydrogen peroxide and other reactive species.

Our results are in harmony with previous studies, demonstrating higher levels of MDA in GC patients [35]. Furthermore, cancer cells may exploit some elements of the oxidative/anti-oxidative systems to sustain survival [36].

Also a previous study showed increased NO levels in GC patients, indicating increased OS in these patients [37]. In addition, an increase in NOS2 activity have been observed in chronic gastritis with IM [38]. Furthermore, NO production is associated with interleukin-6 up regulation and inflammation in gastritis patients and a higher risk of GC [39]. Our findings on protein carbonyl (PC) levels align with earlier studies that have shown increased protein oxidation in GC patients [40] and are consistent with the fact that exposure to pesticides could cause PC induction [41].

The carcinogenic potential of OCPs could be driven by mechanisms such as promoting angiogenesis and cell proliferation, as demonstrated with DDT [42]. Moreover, it has been postulated that β-HCH could increase the aggressiveness of breast cancer cell lines [43]. Exposure to DDE could have significant consequences for the survival of breast cancer cells [44]. Although these specific OCPs have not yet been studied in relation to GC cells, OCPs are known to disrupt the endocrine system, potentially leading to hormone-related cancers, including GC [45]. This is consistent with our findings and previous research, which have documented the increased risk of GC associated with OCP exposure [16]. previous studies in the studied area in Kerman province showed that the proportion of OCPs in patients with breast cancer [17], colorectal cancer [18], bladder cancer [19], thyroid cancer [23] and farmers [22] is higher than control groups.Therefore these findings may indicate the significance of these compounds in the pathogenesis of IM and GC. Additionally, since IM could develop into GC [46] and there are no robust differences in OCP levels between these groups, we may say that for the development of IM into GC, OCPs could just be the tumor promoters, and some more additional genomic changes are needed [26]. Furthermore, since OCPs accumulate slowly in a time-dependent manner in the human body, age could be a major determinant for OCP levels in FD patients (as shown in Table 1) [47]. Moreover, the direct correlation between OCP levels and oxidative stress along with the inverse relationship with AchE, TAC, and antioxidant enzymes, underscores the role of OCPs in inducing OS [48]. Our results are in harmony with the previous study showing the potential of pesticides to induce OS [49]. Given that FD can progress to IM and IM can evolve into GC [46], the observed increase in OCP levels, OS markers, and the decline in antioxidant enzyme activity from the control group to FD, IM, and GC patients suggests that OCP-driven OS may contribute to disease progression toward malignancy [50].

**Table 1:** Age and lipid profiles have been compared between GC, IM, FD and normal Ctrl groups.

Parameter	Groups									
	**GC (n = 34)**	**IM (n = 8)**	**FD (n = 48)**	**Ctrl (n = 46)**	***p*-value (GC vs IM)**	***p*-value (GC vs FD)**	***p*-value (GC vs Ctrl)**	***p*-value (IM vs FD)**	***p*-value (IM vs Ctrl)**	***p*-value (FD vs Ctrl)**
	**Mean ± SD**	**Mean ± SD**	**Mean ± SD**	**Mean ± SD**						
**Age(years)**	63.82 ± 16.29	70 ± 9.52	41.64 ± 17.95	51.69 ± 14.24	0.759	**0.000**	**0.006**	**0.000**	**0.017**	**0.015**
**HDL-c(mg/dl)**	32.73 ± 12.79	26 ± 10.95	37.98 ± 13.19	46.51 ± 3.29	0.375	0.128	**0.0001**	**0.019**	**0.0001**	**0.001**
**LDL-c(mg/dl)**	82.04 ± 63.25	72.37 ± 65.14	82.93 ± 29	111.67 ± 6.02	0.923	1.000	**0.006**	0.894	**0.048**	**0.003**
**Triglycerides(mg/dl)**	111.32 ± 101.46	85.62 ± 17.05	143.45 ± 82.75	162.74 ± 11.55	0.795	0.189	**0.009**	0.15	**0.028**	0.556
**Cholesterol(mg/dl)**	135.88 ± 74.62	115.5 ± 70.18	148.79 ± 33.82	190.74 ± 11.55	0.672	0.593	**0.0001**	0.233	**0.0001**	**0.0001**

All comparisons were performed by using a Post Hoc test. Accordingly, *p*-values less than 0.05 have been implicated as significant.

HDL-c: High-density lipoprotein cholesterol; LDL-c: Low-density lipoprotein cholesterol; v: versus; GC: gastric cancer patients; IM: intestinal metaplasia along with chronic gastritis patients; FD: functional dyspepsia patients; Ctrl: control group.

**Table 2:** comparison of the OCPs serum levels between GC, IM, FD and Ctrl groups.

OCPs(ng/mL)	Groups									
	**GC (n = 34)**	**IM (n = 8)**	**FD (n = 48)**	**Ctrl (n = 46)**	***p*-value (GC vs IM)**	***p*-value (GC vs FD)**	***p*-value (GC vs Ctrl)**	***p*-value (IM vs FD)**	***p*-value (IM vs Ctrl)**	***p*-value (FD vs Ctrl)**
	**Mean ± SD**	**Mean ± SD**	**Mean ± SD**	**Mean ± SD**						
**α-HCH**	0.619 ± 1.25	0.96 ± 1.38	0.56 ± 0.29	0.35 ± 0.54	0.69	**0.01**	0.438	**0.017**	0.187	0.275
**β-HCH**	1.17 ± 5.1	35.29 ± 98.48	1.14 ± 5.87	0.15 ± 0.24	**0.001**	1.000	0.997	**0.001**	**0.001**	0.997
**γ-HCH**	10.48 ± 33.96	7.25 ± 12.03	0.14 ± 0.33	0.96 ± 0.14	0.964	**0.041**	**0.042**	0.701	0.699	1.000
**2,4 DDE**	2.31 ± 5.31	2.77 ± 3.71	0.45 ± 1.15	0.4 ± 0.79	0.978	**0.026**	**0.022**	0.162	0.151	1.000
**4,4 DDE**	0.44 ± 1.16	0.21 ± .0.29	0.33 ± 0.82	0.28 ± 0.68	0.9	0.934	0.835	0.984	0.997	0.993
**2,4 DDT**	2.31 ± 5.14	1.19 ± 0.81	1.39 ± 2.19	0.41 ± 0.87	0.766	0.511	**0.025**	0.998	0.9	0.366
**4,4 DDT**	2.88 ± 5.37	2.05 ± 3.68	2.19 ± 5.81	0.2 ± 0.51	0.964	0.9	**0.045**	1.000	0.705	0.143

All comparisons were performed by using One-Way ANOVA test. As shown differences between groups have been indexed here and *p*-values less than 0.05 are considered significant. The results have been implicated as mean ± SD.

Vs = versus; GC: gastric cancer patients; IM: intestinal metaplasia along with chronic gastritis patients; FD: functional dyspepsia patients; Ctrl: control group.

**Table 3:**
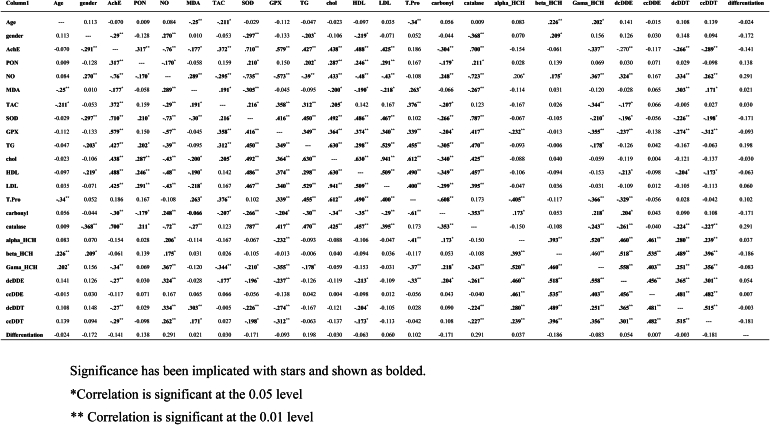
The overall correlation between parameters in OCPs exposed individuals.

This study is the first to measure serum levels of seven OCPs (α-HCH, β-HCH, γ-HCH, 2,4-DDT, 4,4-DDT, 2,4-DDE, and 4,4-DDE) in patients with GC, IM, and FD and to survey the associations of these organochlorine pesticides with the induction of OS in these diseases in the general population of southeastern Iran. However, despite the novelty of our findings, the study faced some limitations, including a small sample size, particularly in the IM group. We suggest an examination of the presence of other types of OCPs in GC patient serums, and also a survey of the OCPs and their association with OS induction in GC and IM patients of other ethnicities. Furthermore, surveys of global histone modifications and tumor suppressor genes promoter methylation status could guide us through the epigenetic mechanisms by which OCPs promote carcinogenesis. Additionally, these findings could serve as a basis for future studies aiming to explore targeted interventions that mitigate oxidative stress and restore enzyme activity, potentially slowing or preventing the progression of early gastric conditions to malignancy. Understanding these mechanistic links also opens pathways for improved diagnostic and preventive strategies in gastric cancer management.

## 5 Conclusion

In our results, progression from FD towards IM and GC was associated with elevation of OCPs, increasing OS parameters (except MDA) and decrease in the activity of AchE and anti-oxidant enzymes in the serum of patients. These facts may indicate the association between serum OCP elevations and the induction of OS in these patients.

## CRediT authorship contribution statement

**Gholamreza Asadikaram:** Methodology, Funding acquisition, Conceptualization. **Mohammad Reza Ashrafi:** Writing – review & editing, Writing – original draft, Validation, Methodology, Investigation. **Sodaif Darvish Moghaddam:** Writing – review & editing, Visualization, Supervision, Project administration. **Moslem Abolhassani:** Writing – review & editing, Software, Resources, Formal analysis, Data curation. **Fatemeh Bagheri:** Writing – review & editing, Resources, Formal analysis, Data curation.

## Consent to participate

All patients who participated in this study were informed about the project and willingly signed the informed consent form**.**

## Data availability statement

Data will be made available on request.

## Ethics approval

Written informed consent was obtained from all participants before the questionnaires were completed and the blood and tissue samples were collected. The Declaration of Helsinki was observed in this study as the cornerstone document for instructing physicians and participants based on standards.

The Research Ethics Committee approved this study of Kerman University of Medical Sciences (Ethics Code: IR.KMU.REC.1398.335).

## Consent for publication

All authors declare their consent to the publication of this paper.

## Declaration of generative AI in scientific writing

The authors declare that they have not used any AI-generated technology in the writing of this paper.

## Funding

This research was financially supported by 10.13039/501100004621Kerman University of Medical Sciences, Kerman, Iran, through Grant No. 96001011. This research was part of a Ph.D. thesis.

## Declaration of competing interest

The authors declare that there is no conflict of interest.
